# Research Progress of Microneedles in Vaccine Delivery

**DOI:** 10.2174/0109298673336874241129081141

**Published:** 2025-01-24

**Authors:** Xinyu Qiao, Derun Liu, Wentao Pan, Meilin He, Fanda Meng

**Affiliations:** 1 Shandong Medicine and Health Key Laboratory of Laboratory Medicine, Department of Clinical Laboratory Medicine, The First Affiliated Hospital of Shandong First Medical University & Shandong Provincial Qianfoshan Hospital, Jinan, 250000, China;; 2 School of Clinical and Basic Medical Sciences, Shandong First Medical University & Shandong Academy of Medical Sciences, Jinan, 250000, China;; 3 Yantai Key Laboratory of Nanomedicine & Advanced Preparations, Yantai Institute of Materia Medica, Shandong, 264000, China

**Keywords:** Microneedles, vaccine, transdermal, immunization, epidemics, MN-based vaccines

## Abstract

Large-scale infectious diseases have become a significant threat to human health and safety. The successful invention of vaccines is the most powerful means for preventing infectious diseases and has greatly improved global human health. Even during the pandemic of COVID-19, which has affected the world, vaccines have played an irreplaceable role. Microneedles (MNs) punctured the stratum corneum and formed microchannels in the skin allowing the vaccine to be efficiently recognized by the abundant antigen-presenting cells (APCs) in the skin to form specific immunity. Compared with traditional vaccination methods, MN transdermal immunization has the advantages of painlessness, easy storage, and efficient immune response. In this review, we summarize the types of vaccines, types of MNs, research progress and clinical research status of MN-based vaccines. We also cover various technologies for vaccine encapsulation, stable delivery of MN vaccines, and a wide range of potential clinical applications. We also outline the future development prospects of the MN system onboard to achieve better clinical benefits.

## 1 INTRODUCTION

Microneedles (MNs) are a novel transdermal drug delivery device consisting of an array of fine needles ranging in height from 10 to 2000 μm and width from 10 to 50 μm. It can penetrate the epidermal layer of the skin into the dermis without touching the nerve and blood vessels, thus causing no pain [1]. At present, MNs hold significant prominence in the field of biomedicine, including drug delivery, biological testing, biomarker extraction, and vaccine immunization and other applications [2, 3], as shown in Fig. (**1**). This minimally invasive and portable drug delivery device has attracted great research interest as an alternative to the hypodermic needle. In addition, MNs are mostly prepared from natural or synthetic polymers with good biocompatibility. In the application process of drug administration, the polymeric needle-tips would dissolve or degrade in the skin tissue, creating almost no medical waste like traditional injection needles. Gerstel and Place first proposed the concept of MNs as a drug delivery system in 1971 [4]. However, MNs were not truly utilized for transdermal drug delivery until 1998. After 2000, the number of studies reporting on MNs has increased rapidly. In 2010, dissolving MNs were used as a biological product to carry vaccines [5], and then the phase I trial of MNs for trivalent influenza hemagglutinins, which involved 40 healthy subjects, was carried out in 2015 [6].

Generally, infectious diseases are defined as diseases spread by specific pathogenic microorganisms or parasites [7]. Infectious diseases have consistently been a public health problem plaguing the world, frequently manifesting as epidemics and a variety of daily infections that affect the population. As the most revolutionary and important invention, vaccines play an important role in human resistance to infectious diseases. The development and administration of vaccines have also significantly mitigated the spread and impact on global health. According to the World Health Organization (WHO), vaccines can prevent more than 2.5 million child deaths globally each year [8]. The vaccines can enhance the immune response of the body, enabling it to effectively combat specific pathogens. The vaccine preparations commonly used in clinical practice can be roughly divided into three categories, namely whole-pathogen vaccines, subunit vaccines, and nucleic acid vaccines. There are still some innovative vaccines in the research and development stage, such as recombinant vectors, which are similar to pathogenic microorganisms and can induce immune responses.

To date, most vaccines have been administered by subcutaneous or intramuscular routes. However, the poor compliance and intense pain caused by subcutaneous or intramuscular vaccination make it difficult to administer the vaccine, which requires trained professionals to administer the vaccine. This has undoubtedly increased the medical burden. In addition, unexpected circumstances, such as contaminated needles, can escalate the risk of disease transmission. As bioactive agents, the strict storage conditions required for cold-chain transport pose a major barrier to the widespread dissemination of vaccines, especially in developing countries [9]. Insufficient supply or production constraints could also be potential problems when there are large vaccine demands. Each type of injectable vaccination relies on the presence of DCs in the tissue that can present antigens to T lymphocytes. However, subcutaneous adipose tissue and muscle tissue contain fewer DCs than the dermis and epidermis, so traditional injectable delivery of vaccines bypasses the immune cell population in the skin, resulting in low immune efficiency [10]. Transdermal drug delivery is defined as the delivery of drugs through the skin, serving as a local or systemic route to achieve clinical approval for use [11]. According to these reasons, transcutaneous immunization is a promising method of vaccine delivery that could induce stronger immune responses and reduce the dosage of administration [12].

So far, many researchers have tried to carry various drugs and vaccines in MNs, aiming to enhance transdermal delivery efficiency, drug utilization, and immune response. At present, the technology of vaccines mounted on MNs is gradually developing. Different kinds of vaccines are mounted on MNs through specific processes to achieve immune effects. In this review, we summarize the development of MNs, the types and materials in vaccines and the efficiency factors, focusing on the current research progress of MN vaccines. In addition, we also summarize the clinical research progress and present our brief introduction.

## 2 TYPES OF VACCINES AND THEIR MECHANISMS OF ACTION

At present, vaccines can be broadly categorized into six different types, as shown in Fig. (**2**), which are live-attenuated, inactivated, recombinant vector, nucleic acid, subunit and toxoid vaccines, respectively, each differing in their sources, components, and immunogenicity.

Live-attenuated vaccines, one of the first vaccines to be developed, successfully eradicated smallpox and had a dramatic impact on polio and measles worldwide [13, 14]. It has been reported that the example of Rinderpest, which occurred in Kenya in 2001, is the most successful live-attenuated vaccine so far [13]. Noticeably, it is the use of live-attenuated vaccines in mass campaigns that transmission of the wild-type poliovirus was broken [15]. These vaccines are often convenient for providing long-term immunity and can stimulate a more comprehensive immune response, including humoral immunity, secreted antibodies, and so on [16]. Acknowledged as one of the most efficacious health interventions available, live-attenuated vaccines against viral diseases are highly regarded [13]. Although this type of vaccine is the only one that does not require additional adjuvants, it requires appropriate cold chain conditions for storage, and its safety is also related to the virulence of the strain [17, 18].

Inactivated vaccines are prepared after the pathogen is inactivated by treatment such as radiation, which destroys the ability of the pathogen to replicate *in vivo* but retains its immunogenicity [19]. Previous studies have reported that vaccine-specific titers against homologous viruses are sometimes higher after inactivated influenza virus administration compared to live-attenuated influenza virus. Additionally, induction of cross-reactive ADCC-antibodies in serum is better with inactivated influenza virus [20-22]. The safety and immunogenicity of CoronaVac as an inactivated virus COVID-19 vaccine were confirmed in a prospective cohort study conducted at Samut Sakhon Hospital in Thailand [23]. Furthermore, there are three main inactivated virus COVID-19 vaccines that have been authorized for emergency use [24]. However, it is usual that virus inactivation may turn out to be incomplete, so outbreaks occur as a result. Coupled with destroyed viral-neutralizing during inactivation, this will result in poor neutralizing antibody response and poor protection upon challenge.

Toxoid vaccines are prepared from toxins secreted by bacteria. Nowadays, the technology for preparing toxoid vaccines is relatively mature, and the vaccines produced show good stability and immunogenicity. The most successful examples are tetanus vaccines and diphtheria vaccines, which have achieved remarkable results worldwide [25, 26]. Typically, live vaccines are contraindicated during pregnancy due to the potential risk of viremia or bacteremia. However, such vaccines, represented by tetanus toxoid vaccines, are safe during pregnancy [27]. In addition, the stability and immunogenicity of many toxoid vaccines are significantly improved after the addition of specific adjuvants [28].

A recombinant vector vaccine is a kind of vaccine in which the coding sequence of the pathogen antigen is inserted into a vector as a foreign gene to exert an effect [29]. Various viruses, including Adenovirus, cytomegaloviruses, poxviruses, and retroviruses, have been engineered into vectors for this purpose [30-33]. Recombinant vector vaccines are characterized by their robust immunogenicity. Recombinant DNA technology is the key to recombinant vector vaccines. Usually, the disadvantage of such vaccines is that the foreign genes in the vector genome are often mutated, which leads to reduced immunogenicity or even loss of vaccine effectiveness [34, 35].

Subunit vaccines refer to the identification and screening of some subunits and proteins as vaccines to induce protective immunity [16, 36]. In general, the simplest and most basic form of subunit vaccines is to decompose the infectious source or pathogen into its parts and wrap purified envelope glycoproteins on the outside of the subunit to reduce toxic side effects. However, this vaccine often has low immunogenicity [16]. Additionally, subunit vaccines often require booster immunizations as well as adjuvants [33].

In recent years, emerging nucleic acid vaccines, such as DNA or RNA vaccines, are characterized by their ability to efficiently self-amplify or transcribe in host cells after entering the body, making them the focus of vaccine development [37, 38]. Of course, mRNA vaccines have the advantage that they do not induce vector-specific immunity and do not contend with either pre-existing or newly raised vector immunity that could interfere with subsequent vaccinations [33].

To sum up, vaccines serve as crucial immune barrier for the body, and different types of vaccines are also being used in the prevention and treatment of distinct diseases. The evaluation based on immunological knowledge and modern pharmaceutical technology can make the vaccine show the best effectiveness and can provide a good plan in the process of preparation, production, transportation and so on. In numerous countries and regions, vaccination has significantly diminished the occurrence and fatality rates of infectious diseases, with vaccination programs being hailed for pivotal contributions to public health achievements. Nevertheless, it is imperative to acknowledge the pressing necessity for expediting scientific innovation in vaccine technology. In recent years, the problems of vaccine immunization cannot be ignored. Such as shelf-life tracking, prevention of wastage due to temperature excursions, needle-reuse and reducing the risk of bloodborne disease, among other things, make it difficult to achieve broader immunization. As suggested by the new Immunization Agenda 2030 and the Gavi 5.0 strategy, there is an urgent call for vaccine product innovations that effectively tackle immunization barriers and ensure equitable vaccine coverage [39].

## 3 THE ANATOMY OF THE SKIN AND MECHANISMS OF TRANSDERMAL IMMUNIZATION

### 3.1 The Anatomy of the Skin

As the largest organ of the human body, the skin is the first barrier for external substances to enter the human body. It can resist the stimulation and interference of various risk factors in the external environment. Indeed, the skin plays a vital role in immunity due to the presence of numerous types of immune cells within its layers [40].

The skin is composed of three layers: epidermis layer, dermis layer, and subdermal loose connective tissue [41], as shown in Fig. (**3**). The epidermal layer consists of five layers, with the outermost layer, the stratum corneum, rich in dense dead keratinocytes and keratins, providing a complex physical barrier to the skin [9]. The stratum corneum below the epidermal layer contains keratinocytes and pigment-related cells [40]. The dermis is the gathering place of blood vessels, lymph, nerve endings, sweat glands, hair follicles, and other structures, where the cell composition is more complex. The dermis is not only a host layer for functional tissue networks but also provides support for other structures in the skin [42]. The loose connective tissue under the dermis mainly contains fat cells, which can store fat and regulate body temperature and buffer [43]. It can be seen that the three-layer structure of the skin provides a solid physical barrier for the body to resist the interference of various factors in the external environment and becomes an indispensable barrier to protect the human body.

The skin also plays an enormous role in immunity. The skin contains a large number of Langerhans cells, macrophages, special APCs and other immune cells, which can perform functions such as capturing antigens, transporting them to lymph nodes, activating T cells, or directly activating follicular DCs to stimulate or regulate the corresponding immune response [44]. The presence of a strong and dense network of APCs in the skin makes it highly likely that antigens will produce a strong and durable immune response in the skin [16]. It is not difficult to see that the skin, being such a site that can induce a good immune response, has become a target for researchers to study vaccines. Additionally, due to the multiple advantages such as easy administration, less exposure, and controlled release, transdermal drug delivery system has become a promising drug delivery method [45].

### 3.2 The Mechanism and Advantages of MNs Transdermal Immunization

MNs Transdermal immunization refers to a non-invasive form of vaccine administration by MNs, in which antigens and adjuvants are directly delivered into the skin structure, inducing potent specific antibody and cell-mediated immune responses. DCs, as major APCs, initiate major T cell responses that effectively stimulate memory responses to connect different immune systems [46]. It is generally believed that the skin allows only small-molecule drug penetration, and the transdermal delivery of biological macromolecule drugs such as vaccines is limited. MN transdermal immunization can break the stratum corneum barrier and improve the delivery rate of macromolecule drugs. The existing MN technology is a very promising approach for delivering vaccine antigens to the skin.

MN transdermal immunization has shown significant advantages in immunotherapy compared with traditional immunotherapies such as intramuscular injection and subcutaneous injection. Remarkably, the tiny tip structures can greatly reduce the patient's pain perception and thus improve patient compliance. Compared to subcutaneous and muscle tissue with only a few immune system defense cells, there were more antigen-presenting cells in the upper dermis of the skin. MNs can accurately deliver vaccines to skin tissue, which improves vaccine utilization and reduces vaccine doses. These MNs are mostly prepared by dissolving or biodegradable natural or synthetic polymers, thus dissolving and degrading the polymers in a simple application process after implantation into the skin. Therefore, the material of MNs often does not cause environmental pollution and has been almost dissolved in the tissue to achieve a pollution-free effect when it plays its role. Therefore, when facing the problem of medical waste treatment, MNs can greatly reduce the pollution problem through the degradable process.

In addition, MN-based vaccine technology can largely change the vaccine dosage form or use the special adjuvant to provide a favorable microenvironment for the vaccine, thereby reducing cold chain transportation and reducing costs. Most importantly, MN vaccines can greatly improve the immunity. Compared to intramuscular injection, MN-based vaccine delivery can induce a stronger immune response even at lower antigen levels. Ellison *et al.* reported that the VaxiPatch system produced IgG antibody levels 100 times higher than the control group [47]. Using MNs as a vaccine delivery platform can significantly enhance immune efficiency, making low-dose vaccination possible. However, further research is needed to determine the optimal dosage for each vaccine and address biosafety concerns.

## 4 THE DEVELOPMENT OF MICRONEEDLES IN VACCINES DELIVERY

Transdermal immunization of MNs is regarded as a potential alternative to traditional vaccination and has good development and application prospects. MNs consist of an array of micron-sized needles connected to a base layer. The needle lengths could be controlled to penetrate the stratum corneum and form microchannels in the epidermis and dermis without damaging nerves or blood vessels, to achieve the purpose of painless transdermal vaccination [48, 49]. Due to the abundance of APCs in the skin, only a small amount of vaccine antigen is needed to trigger an immune response, which greatly reduces the amount of vaccine used in production. Moreover, it is more notable that the vaccine can be stored in solid or solidified form on MNs, which will ensure the vaccine remains more stable at room temperature [50]. Together with the stabilizing effect of other substances, such as adjuvants, the stability of the vaccine on MNs can be greatly enhanced and the cold chain cost of vaccine transportation can be reduced [51]. Because MNs can be self-administered, medication compliance is better, which could improve the efficiency of vaccination.

The mode of MNs loaded with vaccines can be varied based on the type and nature of the vaccine. One is to coat the surface of the MNs with a layer containing the vaccine. When the coating MNs penetrates into the skin, the vaccine can quickly dissolve into the skin. Another approach is to mix the vaccine into the degradable polymer and make the mixture into the MNs in solid form. When the MNs are inserted into the skin, the mixture dissolves, releasing the vaccine and eliciting the immune response [52], and this action process is shown in Fig. (**4**). In addition, MNs loaded with vaccines can be adjusted based on the type, shape, height, and materials of MNs.

### 4.1 Types of Microneedles in Vaccine Delivery

As mentioned above, MNs have [53] been widely used in transdermal drug delivery systems, and are also being developed for various types of vaccine delivery, which can be roughly divided into solid MNs, coated MNs, hollow MNs, dissolving MNs, and hydrogel MNs (Table **1**).

Solid MNs were first mentioned in 1971. The principle is to insert solid MNs into the skin, and then deliver drugs and other compounds through the channels formed by them. The use of metal, silicon, polymer, and other MN materials has been studied in the past [53]. The commonly used Si MNs can be fabricated by the silicon dry-etching process [52]. Chandbadshah *et al.* used array-scale simulations of solid MNs to show that porous silicon and PEGDA (polyethylene glycol diacrylate), among others, could be used to deliver COVID-19 vaccines and obtain good experimental results [54]. It must be said that although solid MNs are a potential candidate to improve the drug delivery ability of transdermal drug delivery systems, it does possess certain limitations such as non-uniform drug delivery [55], complexities in acquiring pharmacokinetic data [56], micro-pores healing of skin [57], medical waste and so on.

Coated MNs refer to drugs and vaccines being coated on the needle-tips of MNs, which are subsequently released upon penetration into the skin to elicit immune effects. DeMuth *et al.* reported rapid-release MNs coated with DNA vaccine. Polyelectrolyte multilayer materials were used in the preparation process to coat the DNA vaccine with a nanostructured film formed by iterative adsorption of alternately charged polymers. To coat the DNA vaccine on the needle-tips, their team used biotinylated poly(o-nitrobenzyl)-methacrylate-co-methyl-methacrylate-co-poly(et- hylene-glycol)-methacrylate (PNMP) film was used as the release layer and coated on the MN tip by spray deposition from 1,4-dioxane solutions to avoid UV exposure of the DNA vaccine [58]. Furthermore, this MN vaccine also enhanced the generation of memory T cells and related gene expression when compared to traditional intradermal injection. This suggests that this MN vaccine can generate a more luminal immune response over an extended period [58, 59]. Meanwhile, Choi *et al.* also successfully used coated MNs to administer the smallpox vaccine. Choi's team used a micro-molding method to prepare polylactic acid (PLA) MNs with polyvinyl alcohol (PVA) as an excipient, on which the smallpox vaccine was loaded by impregnation coating [60]. Given that coated MNs can destroy the vaccine activity during drying, the formulation of the coating needs to be improved. To ensure the preservation of antigen activity and facilitate the effective administration of the influenza vaccine within MNs packaging, Kim *et al.* employed carbohydrate adjuvants, such as trehalose, for stabilizing inactivated flu vaccines [61]. However, it must be noted that the coated MNs could carry the vaccine with the maximum dose of 1mg, and the coating formulation should be optimized according to different vaccine molecules during storage and application to ensure stability [62]. In addition, the coating on the needle tips inevitably reduces the sharpness, making it difficult to pierce the skin and reducing the penetration rate [63]. However, residual drugs on the needle tips may infect other patients [64].

Hollow MNs with high dose accuracy carry vaccines through a hollow space to the skin under different pressures. Lithography, laser cutting, 3D printing, micro-molding, and other technologies are commonly used to manufacture hollow MNs [65]. After mechanical tests, the strength of MNs can penetrate the skin of the living body without damage. Van *et al.* found that commonly hollow MNs fabricated for pulse heating glass capillaries, micro-machining or micro- fabrication techniques on silicon wafers, which often pulls out capillaries that may suffer from low tip shape reproducibility, low throughput of the production process, and blockage. Therefore, they adopted a novel MN fabrication method based on wet etching of capillaries, which is cheap and easy to scale up. They investigated the application of hollow manganese, produced by the corrosion of molten silica capillaries by HF, in an inactivated poliovirus (IPV) vaccine and showed that the immune response elicited by hollow manganese was successful, comparable to that elicited by a hypodermic needle [66]. In addition, they developed a hollow MN applicator for precise control of time, velocity, and depth [66]. In 2018, Van *et al.* also carried a cationic liposome HPV vaccine in hollow MNs, combined with a newly developed digitally-controlled hollow MN injection system (DC-hMN-iSystem), and showed stronger cytotoxicity and T cell immune responses than conventional subcutaneous injection in mice [67]. Subsequently, hollow MNs were used to inoculate rats with mumps and varicella vaccines [68]. It was found that DCs in the upper dermis were activated and the immune effect was better than that of subcutaneous immunization. The potential of hollow MNs for antigen delivery is great; however, most of them require a pumping device to deliver the vaccine to the skin layer. Nonetheless, some have used automated injection pumps or manually activated integrated systems [69, 70].

Dissolving MNs are needles made of vaccines and biocompatible or degradable polymers in a certain proportion. The drug or vaccine dissolved in the needle tips of the MNs will be released after being penetrated into the skin. Generally, micro-molding, micro-casting, droplet blowing, and other techniques are used to fabricate dissolving MNs [96]. It is very reassuring that there are no sharp residues left after the dissolving MNs act on the skin, thereby eliminating the possibility of environmental pollution and secondary infection caused by recycling [62, 97]. Rodgers *et al.* embedded inactivated Pseudomonas aeruginosa in polymethyl vinyl ether to make a dissolved MN patch. This approach prolongs the release time of the vaccine, thereby enhancing immune efficacy [49]. Edens *et al.* administered the dissolving MNs vaccine by a dry formulation of measles antigen to rhesus monkeys and showed antibody efficacy comparable to that of subcutaneous injection. The antigen solution was directly mixed with a solution containing carboxymethyl cellulose and sucrose, and then loaded into an MN mold to dry and lyophilize for 24 hours [77]. In addition, more researchers have used the dissolving MNs carrying tetanus vaccine and polio vaccine in rats, and the immunogenicity produced by the MNs is not significantly different from that of subcutaneous injection [86, 98]. Canonical HA is also widely used to produce dissolving MNs for vaccine delivery in infectious diseases and cancer immunization [83]. In fact, the most commonly used polymers are polyvinyl acetate, trehalose, carboxymethyl cellulose, chitosan, *etc.* [99]. In addition, Cole *et al.* examined four polymers that could be used to enhance the stability and immunogenicity of DNA vaccines, and they complexed DNA with the cation-delivery peptide RALA prior to incorporation into the soluble matrix, thereby improving transfection efficiency after release from the polymer matrix, with the potential to make dissolving MNs a good delivery system of DNA vaccines [100]. In recent years, with the outbreak of COVID-19, the research and progress of MNs in the SARS-CoV-2 vaccine have gradually increased, especially promoting the application of MNs SARS-CoV-2 vaccine to clinical practice and truly solving problems for medical treatment, as shown in Table **1**. These studies not only have a good immune effect but also provide a more effective means for the resistance to SARS-CoV-2 and also lay the foundation for the further maturity of microneedling vaccine technology [101, 102]. However, at this stage, regulatory agencies have not provided guidance on safety and toxicity criteria for the licensure of MN-related materials. In the manufacturing process of dissolving MNs, the dissolved substances are required to meet the sterile requirements, which will increase the cost. Given the limited size of the MN patch, the vaccine dose is also a self-evident difficulty. Therefore, there is a pressing need to continue optimizing the formulation of MNs to address the issues [49, 100].

Hydrogel MNs, which were first reported in 2012, appeared later than other types of MNs [103]. Hydrogel MNs are composed of swellable polymers, namely cross-linked hydrogels, which can be applied to the environment of skin tissue due to their hydrophilic nature [104]. Remarkably, hydrogel MNs have higher drug loading capacity and adjustable drug release rate because of the crosslinking rate [103]. After the hydrogel MNs are applied to the skin, they can be removed completely, leaving no polymer in the skin. However, if the hydrogel polymer has not been adequately cross-linked during the manufacturing process [105], it may degrade when it comes into contact with drugs and vaccines. Hydrogels must also be designed to minimize the risk of cytotoxicity, which is crucial for vaccine delivery [40, 105]. In addition, the research prospect of hydrogel MNs carrying vaccines is great, but the research is relatively few, which is expected to be further explored [40, 69].

### 4.2 The Material Composition of Microneedles in Vaccine Delivery

Due to advancements in materials chemistry and engineering, the materials used in MN preparation are continually evolving and innovating. Generally speaking, the materials of MNs can be roughly divided into metal materials, inorganic materials, and polymer materials. Metal materials are used to fabricate solid, coated, or hollow MNs, but the effect is affected by physical specifications such as shape and size when applied [106]. A variety of methods and techniques have been used to enhance the antigen coating on the solid MN surface. It is easy to nanopattern the stainless-steel MN surface to improve the hydrophilicity of the MN surface. Similar to metal materials, inorganic materials can also be used in solid, coated, and hollow MNs, but the most alarming problem of inorganic materials is biocompatibility [107]. Polymer materials are the most promising materials, and a variety of polymers, including polylactic acid glycolic acid (PLGA), have been used for the fabrication of MNs. PVA and polyvinylpyrrolidone (PVP) are often used to prepare dissolving MNs [108, 109] because they can be rapidly dissolved in the skin and overcome the biocompatibility problem. However, the hardness of these materials is not as good as that of metal materials, so some complexes can be added to enhance their hardness, such as carboxymethyl cellulose-trehalose, PVP-cyclodextrin and so on [110, 111].

Initially, MNs are made of silicon (Si), which has an anisotropic crystal structure [112]. Si MNs could be finely fabricated and mass-produced, but their biocompatibility and fragility may cause some safety problems [59]. In later developments, metals such as nickel and palladium are strong enough to avoid problems such as fracture and are therefore more suitable for MN production. Micro-molding technology allows for the production of MNs using materials like ceramics, silicon, and glass [113], but most of them are only suitable for experimental purposes. In recent years, saccharides have also been used in the preparation of MNs [114-116]. Maltose is the most commonly used saccharide, others, including mannitol, trehalose, sucrose, *etc.*, are also commonly used in the preparation of MNs. Furthermore, recent research showed that HA MNs that encapsulated polydopamine nanoparticles have the ability of antioxidation, antiinflammation, and efficient angiogenesis induction to accelerate wound healing [117]. The polymers we mentioned above have also become more and more popular materials, such as polylactic acid (PLA), polyglycolic acid (PGA), PVA, *etc.*, have been reported to be used in the preparation of MNs vaccine [118, 119]. It is the use of biodegradable materials that can achieve sustained batch release, and the polymer can be dissolved and absorbed within a certain period. Various pieces of evidence indicate that MNs made of biocompatible materials are suitable for vaccine-carrying [120, 121]. Courtenay *et al.* compared the *in vivo* immune response in a mouse model by comparing novel hydrogel- formed MNs and dissolving MNs for delivery of OVA. Although both elicited IgG responses, the dissolving MN immune effect was more potent in comparison [84]. At present, the relevant supervision agencies must issue the standards for the establishment of production lines. Additionally, detailed studies are required to elucidate the effects of polymer-vaccine interactions and their subsequent effect on human immune responses.

### 4.3 Influencing Factors of Microneedles in Vaccine Delivery

**Fig. (5):**
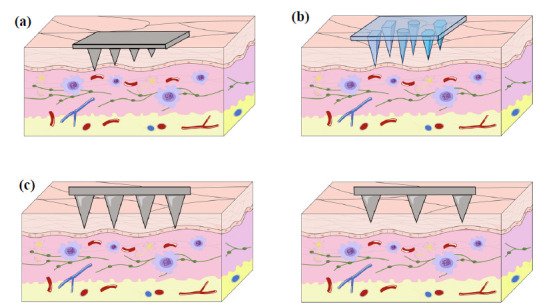
Schematic representation of microneedles structures with different lengths (**a**) Shapes (**b**) and Spacing (**c**).

The effect of the MNs depends on a variety of factors, including the length [122-124], geometry [125, 126], sharpness [60], force [127], spacing [128] of the needles, and so on (Fig. **5**). Typical geometries, including triangles, squares, *etc.*, were confirmed to be superior to hexagons, affecting the insertion depth of the needle tips. In addition, to ensuring proper penetration depth without causing pain, the needle-tip length must be carefully calibrated. The dense array can enhance the formation of microchannels and drug load for drug delivery. However, it requires higher pressure for needle insertion. Studies have shown that the penetration resistance decreased with the increase of spacing, which provides a new idea for optimizing the geometric parameters of MNs [62, 122].

### 4.4 Methods of Vaccine Delivery

There are many ways that solid MNs can be used to deliver vaccines. For example, porous silicon MNs with high porosity can be used for vaccine delivery [129]. However, in the current research work, accurate integrated analysis and simulation of solid MNs have not been carried out, and it is only a hypothetical method and analysis of MNs [54]. In contrast, most solid MNs use a solid needle tip to pierce the skin to form a hole, then remove the solid MNs and apply the drug-loaded formula to the skin [130].

Coated MNs are usually carried out by dip coating [131, 132]. Coated MNs are first treated by the oxygen-plasma method to make their surface more hydrophilic and then dipped multiple times into a coating solution containing vaccine particles. After that, coated MNs are air-dried at room temperature [133]. However, recent studies have demonstrated that coated MNs are more efficient than dissolving MNs in activating APC, so the optimization of coated MNs is further needed [134].

The way the vaccine is delivered by hollow MNs administers the vaccine solution to the skin through the middle pore to exert immune effects.

Dissolving MNs typically have a vaccine solution added to the matrix solution of the MNs to form a mixture of vaccine and matrix, which is then dried and used. For example, to prepare dissolving MNs for HBsAg, the vaccine was added to a PBS solution of maltodextrin and sucrose, followed by fish gelatin and PBS containing sucrose as a substrate to form dissolving MNs [135]. Similarly, IRV-dissolving MNs were prepared by mixing the vaccine in an excipient solution, drying, and then making dissolving MNs [90]. However, there is a lack of experimental quantification of the loss, including the antigen residual on the skin at the time of vaccination, and it is difficult to compare the loss of different ways of carrying vaccines. Moreover, the uneven drug delivery of dissolving MNs has led people to try a variety of pigging-on methods to improve drug delivery.

In recent years, hydrogel MNs have gained increasing popularity, offering diverse methods for vaccine delivery. For example, in hydrogel MNs, long-term vaccine delivery can be achieved in the form of nanoparticle encapsulating vaccines [105, 136]. MNs could help biodegradable nanoparticles to exert their immune effects after entering the skin [130]. Since polymers such as PLGA can form a stable spatial structure to accommodate vaccines when cross-linked, the sustained delivery of vaccines is realized [136]. In 2017, Tu *et al.* developed lipid-coated mesoporous silica nanoparticles loaded with OVA for effective vaccine loading [137]. In addition, many carriers are used in the field of MN drug delivery and are expected to be used for vaccine delivery in the future, for example, metal-based nanoparticles, polymer-based nanoparticles, lipid-based nanoparticles, lipid-coated nanoparticles, and so on [130]. To avoid the deposition of the polymer in the skin, the vaccine contained in the accompanying reservoir can then be permeable and delivered into living skin through a hydrogel structure. Unlike dissolving MNs, the hydrogel MNs can be removed intact from the skin after the vaccine dose has been delivered [84, 103].

### 4.5 Clinical Studies of Microneedles in Vaccine Delivery

At present, MNs can deliver most vaccines that have been tested in mice to verify their safety and immunogenicity (Table **2**). Vaccines against HIV, Ebola, and COVID-19 have good effects on mice [79, 88, 138], which could increase the antibody titer and the survival rate of mice and play an immune-preventive role.

Rouphael *et al.* conducted a phase 1 clinical randomized trial of MNs in which four groups received inactivated influenza vaccine by MN patch, intramuscular injection, autonomous MN patch, or placebo *via* MN patch. The MN patch-loaded influenza vaccine was well tolerated and immunogenic. What is more reassuring is that the MNs have been widely accepted and are more popular than the traditional intramuscular flu vaccine [139]. Several other clinical trials using randomized trials of influenza vaccine with MNs showed no significant differences in effectiveness and immunogenicity of MNs influenza vaccines compared to conventional intramuscular injections [6, 140, 141].

Adigweme *et al.* conducted a double-blind, randomized clinical trial that screened 45 adults (18-40 years), 120 young children (15-18 months), and 120 infants (9-10 months), respectively. Double-blind inoculation of MN patch, subcutaneous injection of measles vaccine, the MN patch placebo, and injection of placebo were used. The results showed that the immune tolerance was high and the acceptance was high in infants and young children. In addition, it is estimated that the cost of the measles vaccine delivered by MNs is less than 60% of the cost of subcutaneous injection, which means MNs delivery is significant in the healthcare system [142].

Similarly, in a single-center study, Debiotech *et al.* randomly assigned volunteers to receive both intradermal and intramuscular doses of rabies vaccine. To determine the safety, tolerability, and immunogenicity of subcutaneous immunization with MNs, we compared them with standard intradermal and intramuscular injections. The results showed a significant reduction in pain with intradermal injection and no significant differences were observed in humoral responses between study routes. Moreover, the safety of the MN delivery device was also verified [143].

In addition, Stephanie's research further validates the preference for intradermal administration, particularly in the context of MN vaccination, which exhibits a heightened level of acceptance among individuals [146]. In the future, a large number of pre-clinical and clinical trials are still needed to confirm the efficiency and convenience of MN delivery vaccines.

In summary, MNs in transdermal immunization show great potential in the development of immunotherapy, and the medical benefits will greatly alleviate social medical problems and provide a better means for the prevention and cure of a variety of diseases.

### 4.6 Possibilities and Limitations for the Application of Microneedle Vaccines

Although the technical development and preparation process of MN vaccines are gradually being optimized and the immune effect is gradually maturing, policymakers and manufacturers need to consider a variety of scenarios when assessing the demand for MN vaccines in order to comprehensively consider the potential market and public health needs. It is clear that the potential public benefits of MN vaccines are clear, such as substantially improving global immunity against infectious diseases, especially in developing countries, and reducing the adverse reactions to vaccination and medical waste contamination [153].

Large-scale commercial production still faces the problem of high cost, especially for MN vaccines with different materials, and the maturity of manufacturing technology needs to be improved to reduce the cost. In addition, a key issue of microneedle vaccine technology is to ensure the stability and efficacy of the vaccine in MNs. Furthermore, more clinical data are needed to verify its performance. At present, the strict approval of drug regulatory agencies around the world is still testing its ability to further develop, but large-scale verification still needs more time [154]. The above-mentioned possibilities and limitations need to be constantly verified and tested, but the degree of public acceptance is still an important factor worth promoting. Whether the public will broadly accept such innovative technologies remains a question, especially in some cultures or regions where acceptance of new technologies may be lower.

## CONCLUSION

In conclusion, MN vaccination offers significant advantages over traditional vaccination modalities. It reduces pain during injections and improves patient compliance. With the improvement of quality of life and the increasing demand for medical services, the continuous progress of vaccine-carrying technology, and biomaterials science, MN vaccines are also being optimized, so the market prospect of MN vaccines is broader and broader. However, the key challenge for the popularization of MN vaccines is to achieve commercial production. Although the fabrication technology of MNs is well developed, large-scale production is still a challenge to overcome. Regulatory authorities must formulate unified quality standards to regulate the production of MN vaccines. At the same time, we must pay attention to the formulation development of MN vaccines in the research and development process to achieve the stability of the vaccine, and many clinical trials are needed to verify its safety in the future. Overcoming these problems will provide a strong guarantee for the development of MN vaccines. With further research, MN vaccines will provide a more convenient and efficient use experience.

## AUTHORS’ CONTRIBUTIONS

The authors confirm their contributions to the paper as follows: draft manuscript and draw figures: XQ, DL, WP; revise and edit: FM, MH. All authors read and approved the final version of the manuscript.

## Figures and Tables

**Fig. (1):**
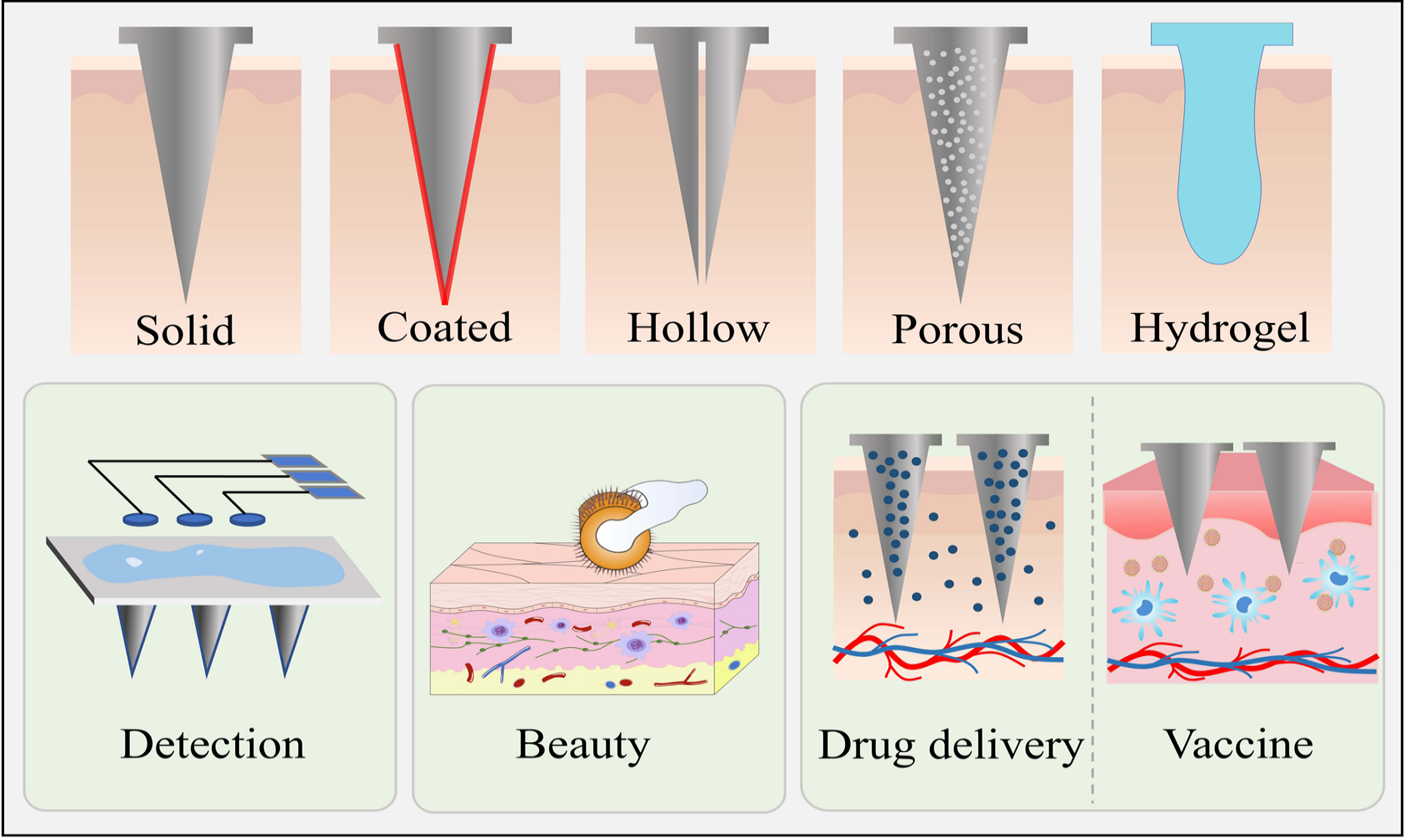
The structure (**A**) and applications (**B**) of microneedles.

**Fig. (2):**
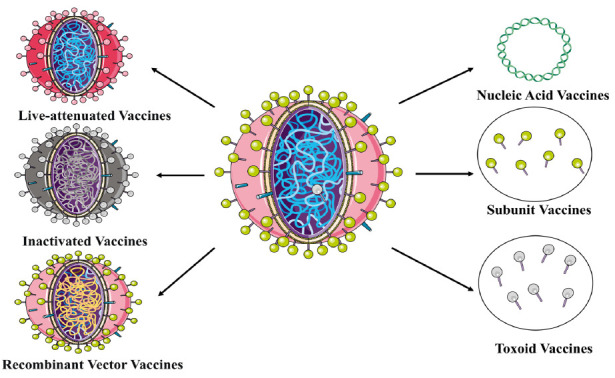
Schematic representation of the six vaccine structures.

**Fig. (3):**
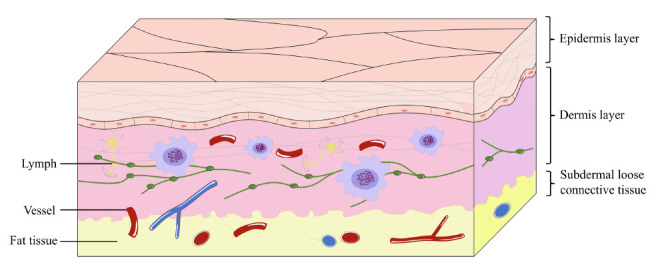
The anatomy of the skin and the structure of epidermis. The anatomical structure of the skin can be roughly divided into the epidermis, dermis and subcutaneous loose tissue. There are no blood vessels in the epidermis, while there are blood vessels, nerves, lymphocytes, and immune cells in the dermis and subcutaneous loose tissue.

**Fig. (4):**
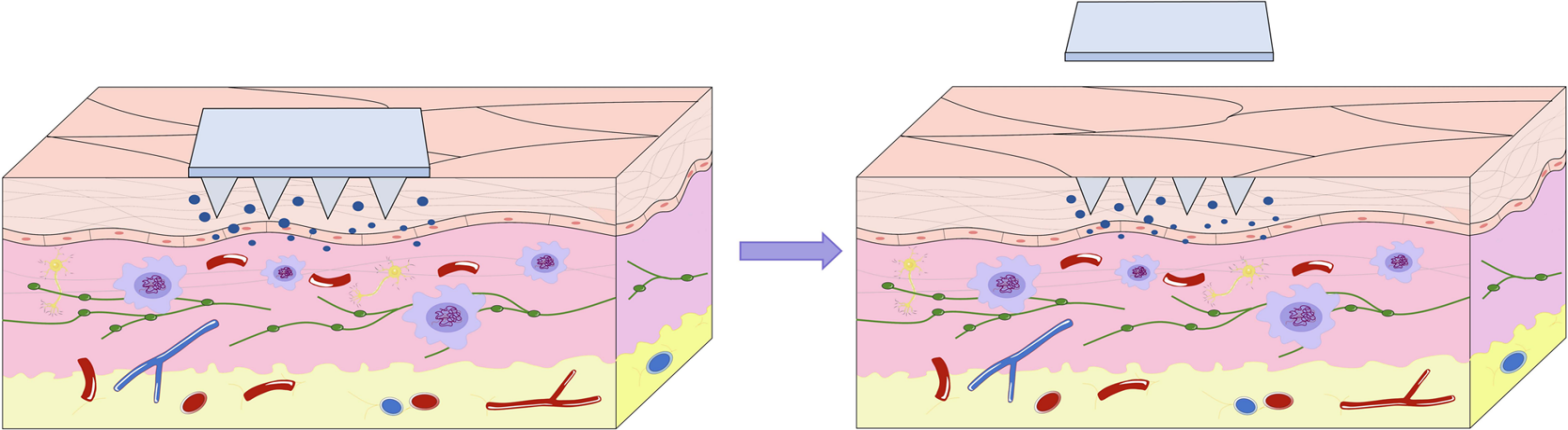
Schematic diagram of the mode of microneedle action on the skin. When the MN patch was applied to the skin, it gradually degraded with time. After the action, the basal layer could be torn off. Since the tip of the needle is only nano-level, it will not damage the blood vessels and nerves, reduce pain and discomfort, and improve autonomy.

**Table 1:** Process of experimental study.

**Time**	**Microneedles Types**	**Vaccine Types**	**Features**	**Citations**
Feb. 2007	Solid (stainless steel) MNs	(i) A whole inactivated influenza virus, (ii) A trivalent split-virion human vaccine, and (iii) A plasmid DNA encoding the influenza virus hemagglutinin	MNs can save doses, and the immune effect is good. MNs can deliver up to 100-fold more than intramuscular injections. The immune response was similar between the two groups.	[71]
Aug. 2010	Dissolving MNs	Influenza vaccination	It can provide a new technology for simpler and safer vaccination with improved immunogenicity that could facilitate increased vaccination coverage. Compared with intramuscular injection, the immune effect of the microneedle vaccine was significantly higher than that of the injection group, and showed a slight advantage.	[5]
Jul. 2011	Solid (silicon) MNs	Recombinant modified vaccinia virus Ankara	It showed that the design of microneedle patches significantly influences the magnitude and memory of vaccine-induced CD8^+^ T cell responses and can be optimised for the induction of desired immune responses. Compared with intramuscular injection, the immune effect induced by the MN vaccine was significantly higher than that by intramuscular injection within 6 hours, such as IL-10 and other cytokines.	[72]
Apr. 2012	Coated MNs	Recombinant adenovirus and modified vaccinia virus Ankara	It is the first report of successful vaccination with recombinant live viral vector vaccines coated on microneedle delivery devices. The MN vaccine patch significantly induced CD8^+^ T cell responses.	[73]
Jul. 2013	Coated MNs	Measles vaccine	Vaccination with MNs at a dose equal to the standard human dose or one-fifth of the human dose resulted in neutralizing antibody levels equivalent to those achieved with the same dose of subcutaneous vaccination.	[74]
Aug. 2014	Solid (silicon) MNs	Adenovirus-based malaria vaccine, HAdV5-PyMSP1_42_	MN-mediated immunization has the potential to both overcome some of the logistic obstacles surrounding needle-and-syringe-based immunization as well as to facilitate the repeated use of the same adenovirus vaccine, thereby potentially reducing manufacturing costs of multiple vaccines. Compared with the injection method, the MN-based method can provide a relatively stable immune expression response with equivalent transgene-specific antibody titer for the malaria vaccine.	[75]
Jun. 2015	Solid (silicon) MNs	Live adenovirus-vectored malaria vaccine	MNs induce CD8^+^T cell responses and protective efficacy. MN vaccines showed the same protective effect as immunization by injection.	[76]
Sep. 2015	Dissolving MNs	Inactivated polio vaccine (IPV)	IPV vaccination using a microneedle patch is immunogenic in rhesus macaques and may offer a simpler method of IPV vaccination of people to facilitate polio eradication.	[77]
Sep. 2015	Dissolving polymeric MNs	Measles virus	It showed that the 12 mIU/ml level was deemed as the titer level needed to confer protection in humans.	[78]
Jan. 2017	Dissolving MNs	Ebola DNA vaccine	The nanoparticle delivery system increases vaccine thermostability and immunogenicity. The immune response of the IgG1 subtype induced by PLGA-PLL/γ PGA MNs was significantly higher than that induced by positive control.	[79]
Apr. 2017	Dissolving MNs	Trivalent subunit influenza vaccine	It showed that optimally formulated MN patches can retain influenza vaccine activity during extended storage outside the cold chain and during other environmental stresses.	[80]
Jun. 2017	Dissolving MNs	M2e vaccine	It proved a rapid approach for increasing the protective efficacy of seasonal vaccines in response to a significant drift seen in circulating viruses.	[81]
Sep. 2017	Coated MNs	Diphtheria toxoid	The layer-by-layer coating approach onto pH-sensitive microneedles is a versatile method to precisely control the amount of coated and dermally-delivered antigen that is highly suitable for dermal immunization.	[82]
Sep. 2018	Dissolving hyaluronic acid (HA) tips and biocompatible polycaprolactone (PCL) bases	Canine influenza virus	It will be particularly attractive for animal vaccinations. The insertion-responsive MNs induced twice as many hemagglutination inhibition antibodies as intramuscular injections.	[83]
Jan. 2019	Hydrogel-forming MNs and dissolving MNs	Protein antigen ovalbumin	It highlights the importance of MN design and the potential impact of dissolving MN polymers on the immune response to vaccine antigens. The data showed that the antibody titers induced by dissolving MNs were significantly higher than those induced by other methods.	[84]
Aug. 2019	Solid pyramidal MNs	HIV envelope trimer immunogen	MNs can substantially enhance humoral immunity to subunit vaccines. The experiment showed that the serum IgG titer in the implantable MN department increased about 1300 times.	[85]
Oct. 2019	Dissolving MNs	Incorporates trivalent inactivated Sabin poliovirus vaccine	MNs can overcome logistic issues currently constraining poliovirus eradication campaigns. The neutralizing antibody responses induced by the MNs were comparable to higher doses of vaccine injected intramuscularly with poliovirus serotypes 1 and 3.	[86]
Oct. 2019	Dissolving MNs	Shigella	It is the first time the potential of outer membrane vesicle-loaded dissolving MNs for ID vaccination against enteropathogens like Shigella	[87]
May. 2020	Dissolving MNs	SARS-CoV-2 S1 subunit vaccines	The results support the clinical development of MNA-delivered recombinant protein subunit vaccines against SARS, MERS, COVID-19, and other emerging infectious diseases.	[88]
Sep. 2021	Nanovaccines-laden separable MNs	COVID-19	The MN patches can be stored at room temperature for at least 30 days without decreases in immune responses.	[89]
Feb. 2022	Combined IRV-IPV dissolving MNs	Inactivated polio vaccine and inactivated rotavirus vaccine	This innovative approach delivered a novel combination vaccine against rotavirus and poliovirus in children throughout the world.	[90]
Mar. 2023	Polyacrylamide/chitosan hydrogel MNs	Ovalbumin	The study was demonstrated to be a low-cost, user-friendly one and displays active delivery, showing great potential for vaccine self-administration at home.	[91]
Dec. 2023	Coated MNs	Split influenza vaccine	It opened up the possibility of eventually obtaining a simple, easy-to-use, and efficient application technology for the prevention of global epidemics like influenza.	[92]
Mar. 2024	Dissolving MNs	S1 subunit protein COVID-19 vaccine	The administration of four doses of the MN vaccine induced robust and long-lasting immune responses, persisting for at least 80 weeks.	[93]
Aug. 2024	Dissolving MNs	Poxvirus vaccine	CSV-loaded DMPs, effectively addressing the storage and transportation challenges, are expected to be utilized worldwide as an innovative technique for poxvirus inoculation, especially in underdeveloped regions.	[94]
Oct. 2024	Dissolving MNs	1D rod-like tobacco-mosaic-virus-based peptide vaccine	By encapsulation of TMV-PEP3 in the tips of a trehalose MN, TMV-PEP3 can be delivered by MN and significantly promote local immune cell infiltration.	[95]

**Table 2:** Process of the clinical study.

**Time**	**Process**	**Microneedles Types**	**Vaccine Types**	**Features**	**Sample Size**	**Citation**
Dec. 2008	phase II clinical trial	BDTM Microinjection System	Influenza vaccines	They offer a less invasive and possibly more acceptable vaccination.	978 healthy adults	[144]
Jan. 2009	phase I clinical trial	Silicon crystal MNs	Influenza vaccines	Low-dose influenza vaccines delivered intradermally using microneedles elicited immunogenic responses similar to those elicited by the full-dose intramuscular vaccination.	180 healthy adults	[140]
Apr. 2014	phase I clinical trial	Stainless steel MNs	Influenza vaccine	Microneedle patches for self-vaccination against influenza are usable and may lead to improved vaccination coverage.	91 healthy adults	[145]
Jun. 2015	phase I clinical trial	NanoPass MicronJet600 MN device	Inactivated Polio Vaccine	A 60% reduction in the standard IPV dose without a reduction in antibody titers is possible through intradermal administration.	231 adults with well-controlled human immunodeficiency virus infection	[146]
Jul. 2015	phase I clinical trial	Dissolving MNs	Trivalent influenza hemagglutinins	The immune effects of microneedle vaccination and routine vaccination were consistent.	40 healthy men	[6]
Aug. 2017	phase I clinical trial	Dissolving MNs	Inactivated influenza vaccine	The use of dissolvable microneedle patches for influenza vaccination was well tolerated and generated robust antibody responses.	100 immunocompetent adults	[139]
Mar. 2020	phase I clinical trial	Coated MNs	A monovalent, split inactivated influenza virus vaccine containing A/Singapore/GP1908/2015 H1N1 haemagglutinin (HA)	The first clinical trial to evaluate the immunogenicity of lower doses of vaccine delivered by microarray patches.	150 healthy adults	[147]
Aug. 2020	phase I clinical trial	Dissolving MNs	Inactivated influenza vaccine	Most participants were accepting IIV vaccination by MNP and preferred it to injection.	100 healthy adults	[148]
Feb. 2022	phase I clinical trial	Low-dose microneedle array (MNA-10%), or high-dose microneedle array (MNA-25%)	Japanese encephalitis vaccine	A microneedle patch of the Japanese encephalitis vaccine is safe, well-tolerated, and immunogenically effective.	39 healthy adults	[149]
Jan. 2024	phase I clinical trial	Dissolving MNs	Synthetic nanoparticle-based, T cell priming peptide vaccine against dengue	Results provide proof of concept that a synthetic nanoparticle-based peptide vaccine can successfully induce virus-specific CD8^+^ T cells. The favourable safety profile and cellular responses observed support further development.	26 healthy adults	[150]
May. 2024	phase I/II clinical trial	Dissolving MNs	Measles and rubella vaccine	All local reactions were mild. There were no related severe or serious adverse events.	age de-escalation healthy person (45 adults, 120 toddlers, and 120 infants)	[151]
Jun. 2024	phase I clinical trial	Dissolving MNs	H1N1 influenza vaccine	The MN platform is safe, well tolerated and elicits robust antibody responses.	45 healthy adults	[152]

## LIST OF ABBREVIATIONS

MNs: Microneedles
APCs: Antigen-Presenting Cells
PLA: Polylactic Acid
PVA: Polyvinyl Alcohol
IPV: Inactivated Poliovirus
PLGA: Polylactic Acid Glycolic Acid
